# Supplementary material for: Human cell surface-AAV interactomes identify LRP6 as blood-brain barrier transcytosis receptor and immune cytokine IL3 as AAV9 binder
Source: Nat Commun. 2024 Sep 8;15:7853. doi: 10.1038/s41467-024-52149-0 (PMC11381518; doi:10.1038/s41467-024-52149-0)
Supplement: Supplementary file 1 — Supplementary Information [file 41467_2024_52149_MOESM1_ESM.pdf]

Supplementary information for

Human cell surface-AAV interactomes identify LRP6 as blood-brain barrier transcytosis receptor and immune cytokine IL3 as AAV9 binder

Timothy F. Shay#*, Seongmin Jang#, Tyler J. Brittain#, Xinhong Chen#, Beth Walker, Claire Tebbutt, Yujie Fan, Damien A. Wolfe, Cynthia M. Arokiaraj, Erin E. Sullivan, Xiaozhe Ding, Ting-Yu Wang, Yaping Lei, Miguel R. Chuapoco, Tsui-Fen Chou, Viviana Gradinaru*

#Authors contributed equally
*Correspondence to: tshay@caltech.edu and viviana@caltech.edu

Contents:

Supplementary Tables 1-4
Supplementary Figures 1-11

Supplementary Table 1. Doses of individual vectors within the pools used to optimize signal to noise

Vector name	Pool dose (v.g. / cell)		
	**7.9 x 10^5**	**5.1 x 10^5**	**1.6 x 10^5**
AAV9	2 x 10^5	1 x 10^5	3 x 10^4
AAV.CAP-B22	6 x 10^4	1 x 10^5	3 x 10^4
AAV-MaCPNS1	1.5 x 10^5	1 x 10^5	3 x 10^4
AAV-MaCPNS2	1.5 x 10^5	1 x 10^5	3 x 10^4
AAV.CAP-Mac	2 x 10^5	1 x 10^5	3 x 10^4
AAV9-X1.1	3 x 10^4	1 x 10^4	1 x 10^4

Supplementary Table 2. Summary of all detected BS3 cross-links with XlinkX score above 40, indicating high confidence, in human IL3-Fc with AAV9 sample

Site 1	Site 2	XlinkX Score
IL3 K85	AAV9 K557	112.8
IL3 K85	AAV9 K462	75.4
AAV9 K462	AAV9 K557	117.89
AAV9 K545	AAV9 K557	68.5
IL3 K85	IL3 K129	50.9

Supplementary Table 3. Chimeric AAV9 capsid amino acid substitutions to AAV8 identity

	**ΔhIL3.1**	**ΔhIL3.2**	**ΔhIL3.3**	**ΔhIL3.4**	**ΔhIL3.5**	**ΔhIL3.6**
Variable region	VR-I	VR-V	VR-VI	VR-VII	VR-VIII	VR-IX
AAV9 positions	AA268 - AA269	AA504 - AA510	AA529 - AA532	AA547 - AA557	AA582 - AA591	AA709 - AA712
AAV9 identity	SS	PGASSWA	EGED	GTGRDNVDADK	TNHQSAQAQA	NNVE
AAV8 identity	AT	TAGTKYH	DDEE	NAARDNADYSD	DNLQQNTAP	TSVD

Supplementary Table 4. Data collection and processing parameters for AAV9-hsIL3, related to Figure 3d and Supplementary Figure 5

Magnification	33,000x
Voltage (keV)	300
Energy Filter	Yes
Slit width (eV)	20
Pixel size (Å)	2.65
Defocus range (μm)	-2.0 to -4.0
Defocus step (μm)	0.5
Tilt range (min/max, step)	-60/60˚, 2˚
Tilt scheme	Dose-symmetric
Total dose (electrons/Å^2)	~60
Frame number	8
Tomograms used/acquired	46/51
AAVs picked	2,661
Map resolution (I1 refined whole)	11 Å

Supplementary Figure 1. Individual characterization of pool AAVs prior to full screen

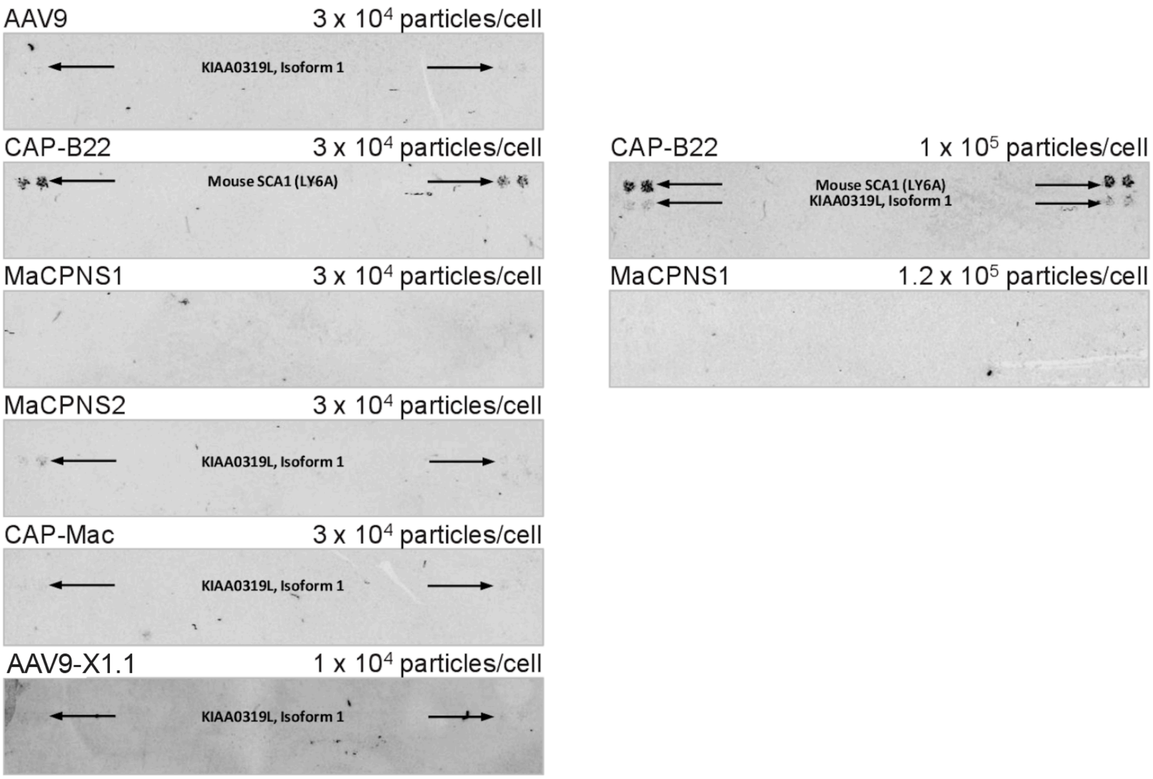

Supplementary Figure 1. Individual characterization of pool AAVs prior to full screen. Individual AAVs were tested at various doses to determine the optimal signal to noise ratio for each capsid while confirming detection of known interactions with KIAA0319L (AAVR) and, for CAP-B22 only, LY6A. AAV binding detected at duplicate spots of the same protein is indicated by arrows.

Supplementary Figure 2. Cell culture potency assay validation of high-throughput screen hits

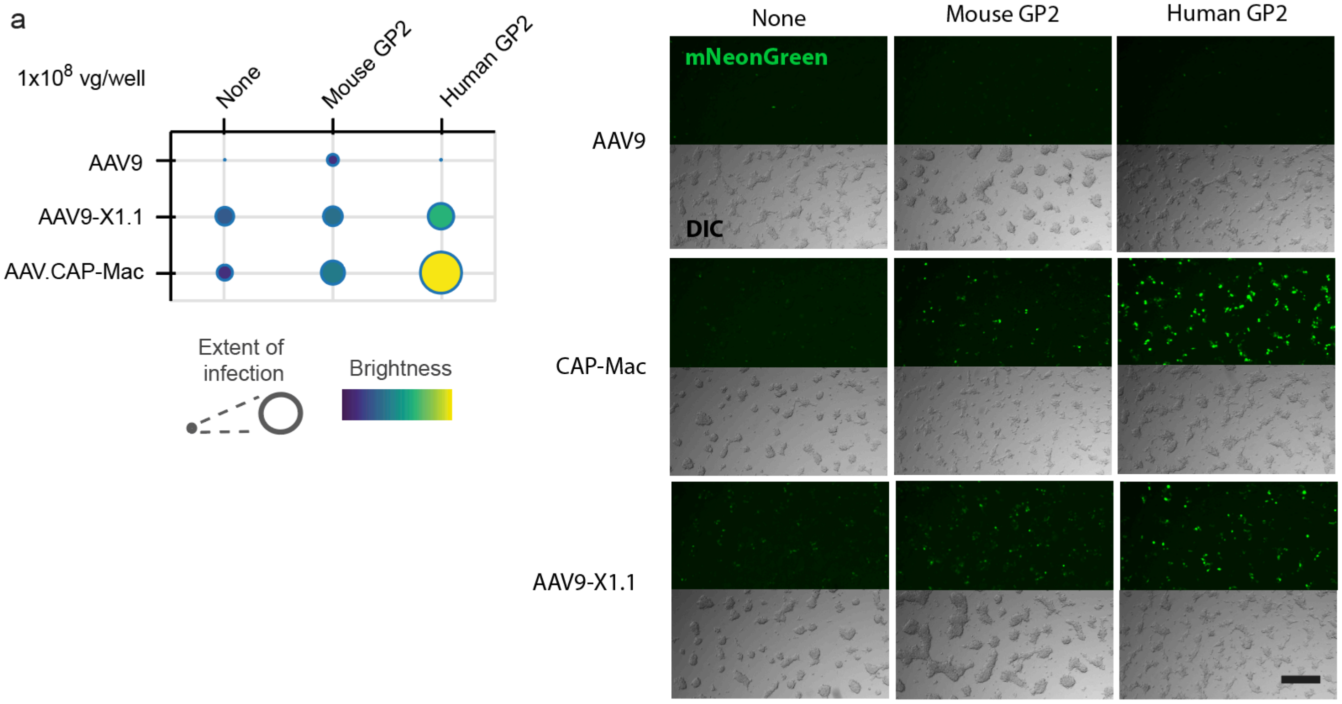

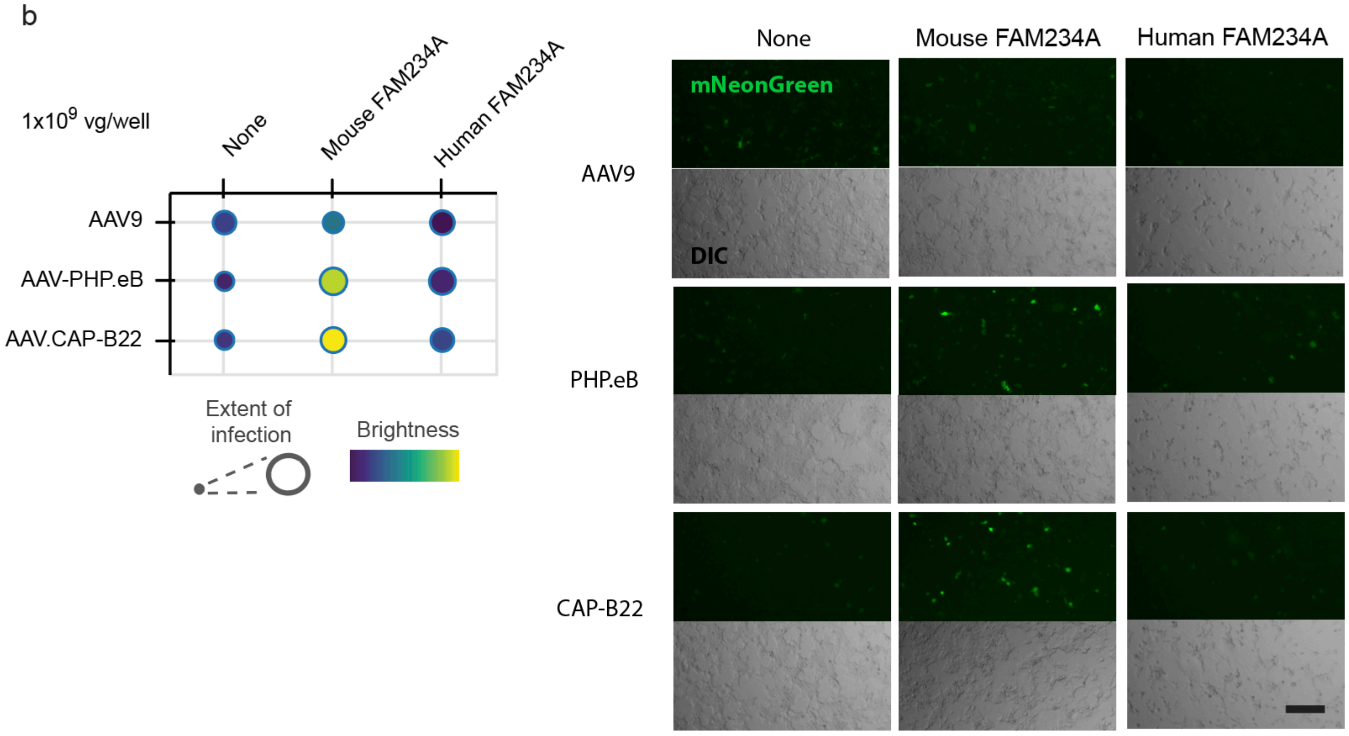

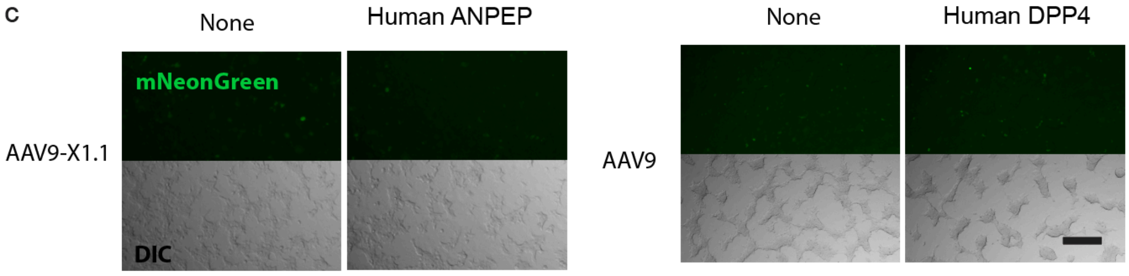

Supplementary Figure 2. Cell culture potency assay validation of high-throughput screen hits. a Transient overexpression of mouse and human GP2 in HEK293 cells resulted in enhanced potency of CAP-Mac and AAV9-X1.1, with a stronger effect for the human protein. Scales show extent of infection (min, 0.01; max, 0.17) and total brightness per signal area (min, 0.11; max, 0.45). Data were quantified from 3 biological replicates. **b** Transient overexpression of mouse and human FAM234A in HEK293T cells results in enhanced potency of PHP.eB and CAP-B22, with a stronger effect for the mouse protein. Extent of infection (min, 0.04; max, 0.07) and total brightness per signal area (min, 0.16; max, 0.29). **c** Transient overexpression of human ANPEP and DPP4 did not result in potency enhancements for AAV9-X1.1 and AAV9, respectively. Scale bars indicate 200 μm. Data were quantified from 3 biological replicates. v.g.: viral genomes. DIC: differential interference contrast.

Supplementary Figure 3. SPR confirmation of selected screen hits

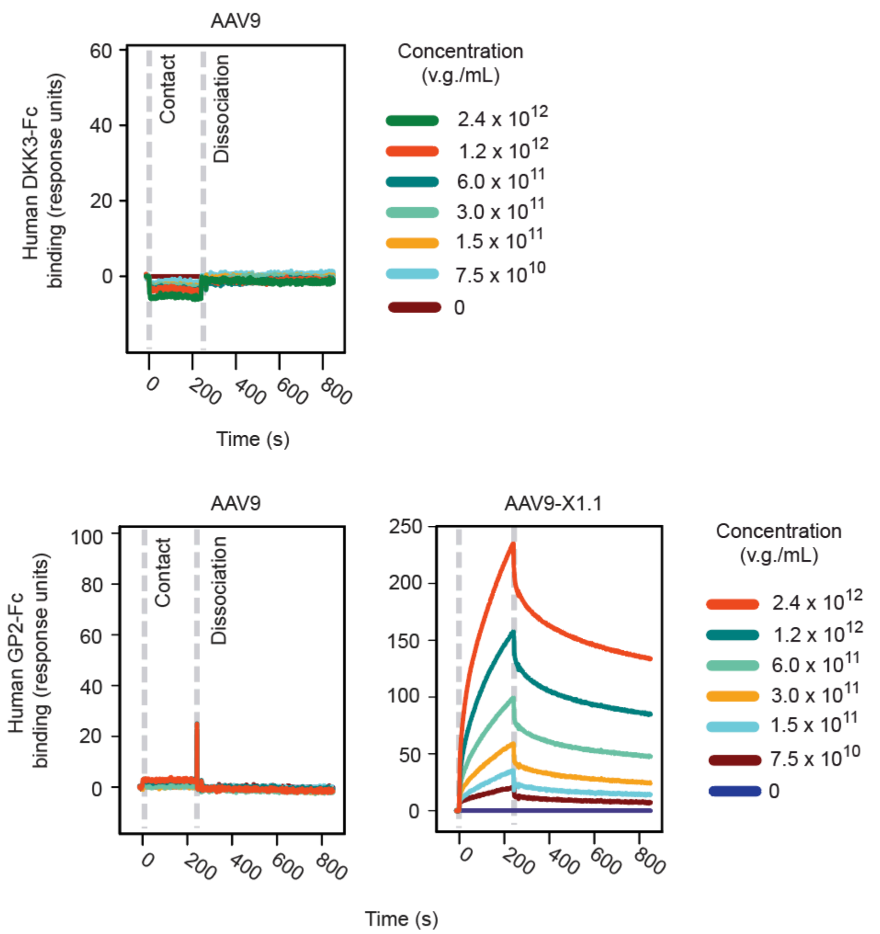

Supplementary Figure 3. SPR confirmation of selected screen hits. Immobilization of human DKK3-Fc or human GP2-Fc on a protein A chip allowed AAV analyte interactions to be assessed. In contrast to the cell microarray screen, no interaction was observed for AAV9 with DKK3, whereas AAV9-X1.1 gained direct binding to human GP2, in agreement with the cell microarray screen and cell culture potency assay. v.g.: viral genomes.

Supplementary Figure 4. Cross-linking Mass Spectrometry confirms interaction between AAV9 and human IL3

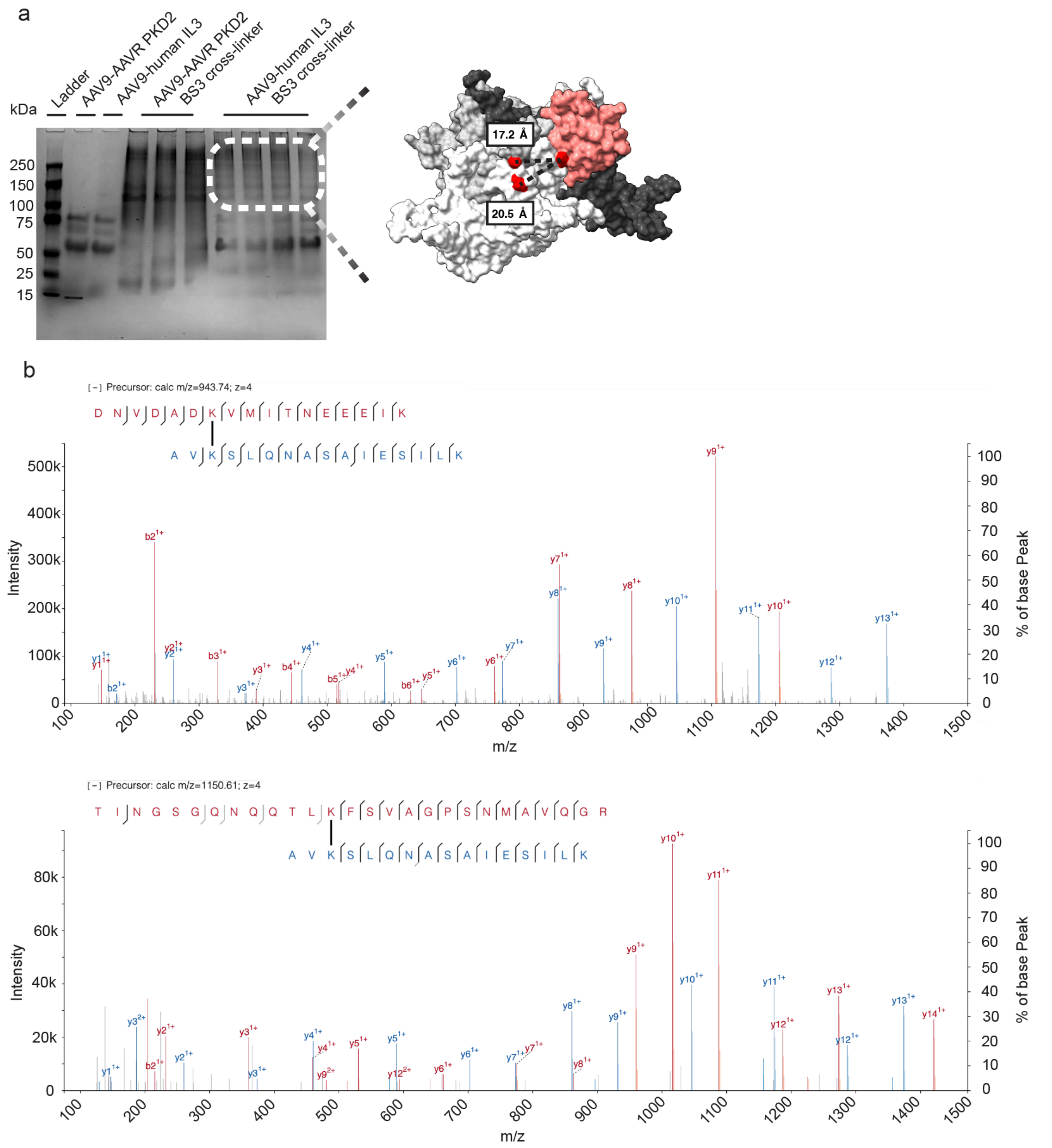

Supplementary Figure 4. Cross-linking mass spectrometry confirms interaction between AAV9 and human IL3. **a** PAGE gel of BS3 cross-linked AAV9 and human IL3. Dotted boundary indicates the region extracted for MS/MS analysis, revealing two intermolecular cross-links. These cross-links are illustrated for one potential interaction mode generated by rigid docking of human IL3 on AAV9. AAV9 trimer in white, grey, and black. Human IL3 in red. **b** Fragmentation spectra of peptides for each intermolecular cross-link between AAV9 and human IL3 with

XlinkX score[82] above 40, indicating high confidence. Red and blue y and b fragments indicate the peptide of origin presented in the upper left.

Supplementary Figure 5. Surface Plasmon Resonance of AAV9 binding to IL3 chimeras

a

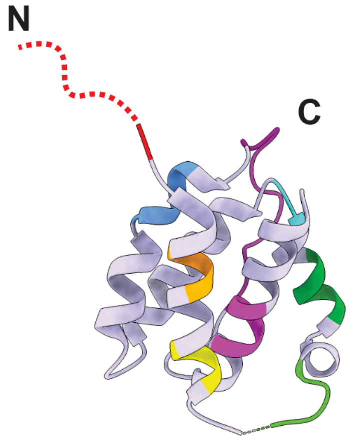

	human IL3 positions	human IL3 AA identity	Chimeric marmoset AA identity
IL3-1	AA21 - AA32	PMTQTTPLKTSW	APTQTMPLKTTQ
IL3-2	AA38 - AA40	MID	LRE
IL3-3	AA45 - AA47	HLK	LLN
IL3-4	AA52 - AA55	PLLD	ASSN
IL3-5	AA61 - AA65	GEDQD	REDQR
IL3-6	AA69 - AA70	EN	KP
IL3-7	AA99 - AA102	NLLP	DLPV
IL3-8	AA106 - AA117	LATAAPTRHPIH	TANAATQHPIR
IL3-9	AA127 - AA131	RRKLT	QMKLK

b

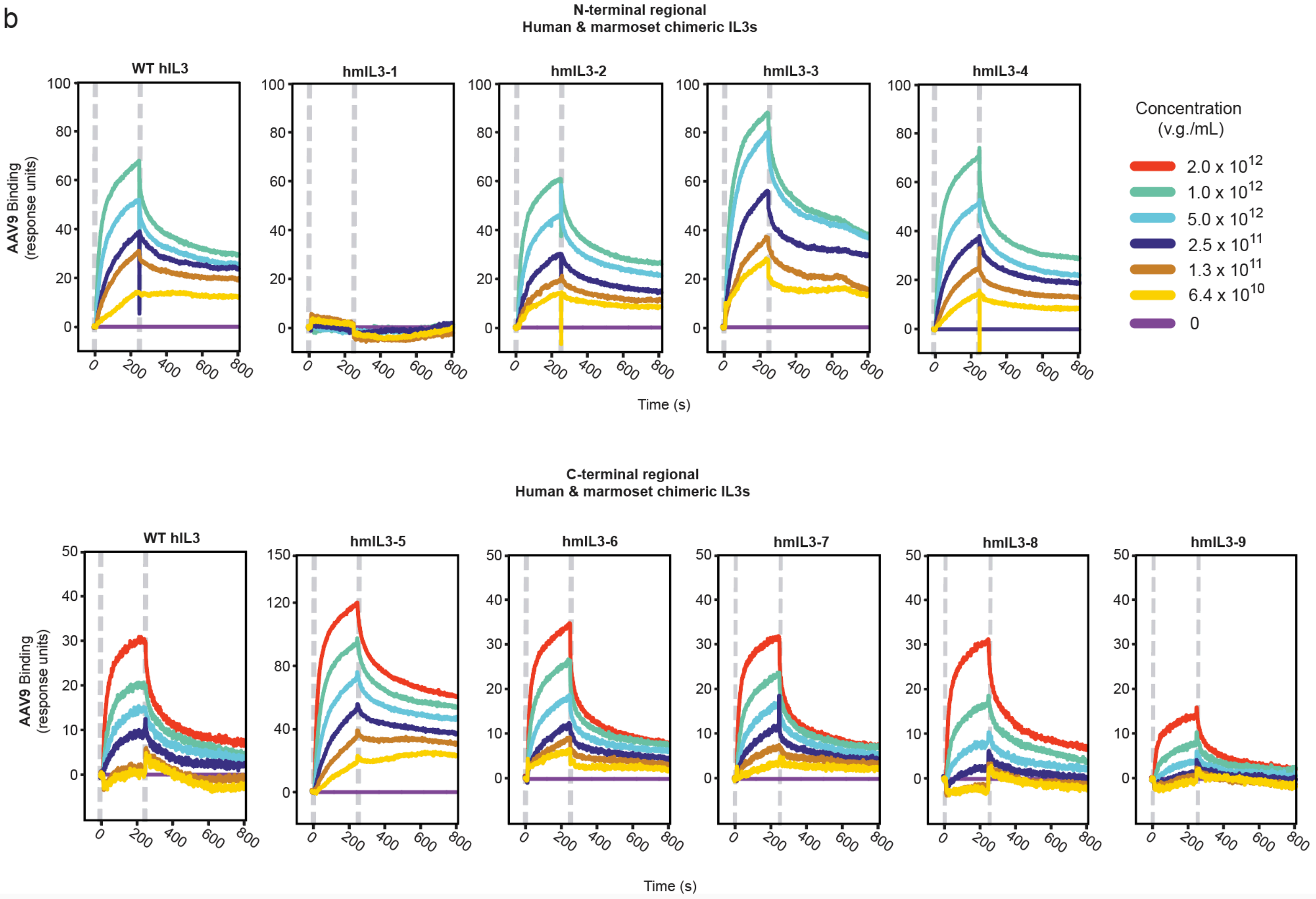

Supplementary Figure 5. Surface plasmon resonance (SPR) of AAV9 binding to IL3 chimeras. a Schematic of human IL3 structure (PDB ID: 5UV8) and regions mutated to marmoset amino acid identity in the chimeras. **b** SPR experiments showing the AAV9 binding capacity of each IL3 chimera. v.g.: viral genomes.

Supplementary Figure 6. Cryo-ET data processing pipeline for human IL3-Fc-bound AAV9

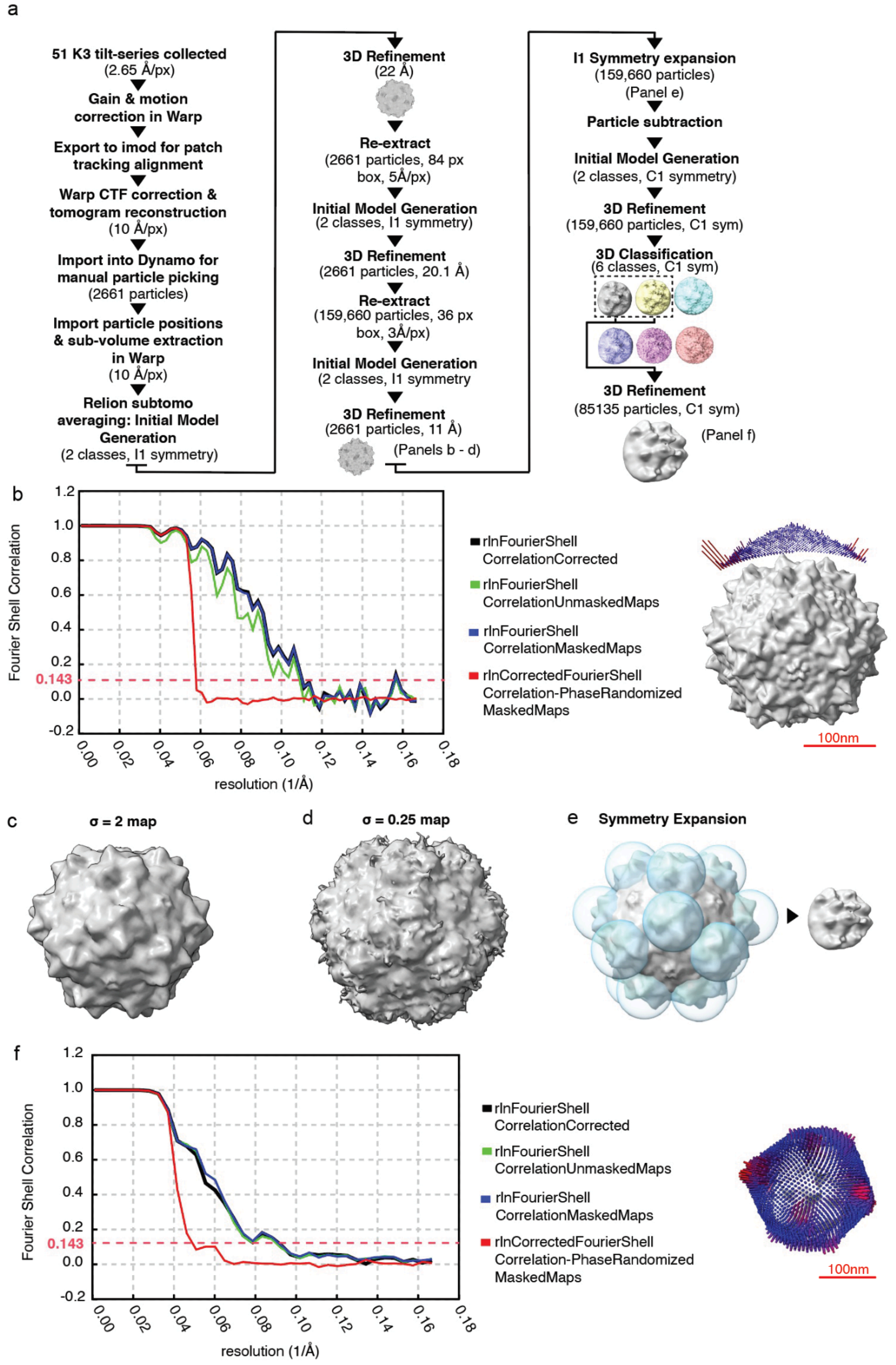

Supplementary Figure 6. Cryo-ET data processing pipeline for human IL3-Fc-bound AAV9. a Schematic of simplified pipeline for cryo-ET data processing in Warp and sub-volume averaging in Relion. **b** FSC plot for the I1-enforced IL3-bound AAV9 map (left) and Euler angle distribution shown overlaying I1 capsid map (right). **c** High-sigma view of I1-enforced IL3-bound AAV9 map at 11 Å resolution. **d** Low-sigma view of same map showing possible human IL3 density. **e** Symmetry expansion and localized reconstruction of the AAV9 trimer resolves the density of human IL3. **f** FSC plot for the symmetry-expanded IL3-bound AAV9 trimer (left), shown for map quality purposes only as we did not actively exclude potential duplicated particles, which can lead to resolution overestimation, and Euler angle distribution shown overlaying 3-fold symmetry face map (right).

Supplementary Figure 7. Integrative binding model of human IL3-Fc-bound AAV9

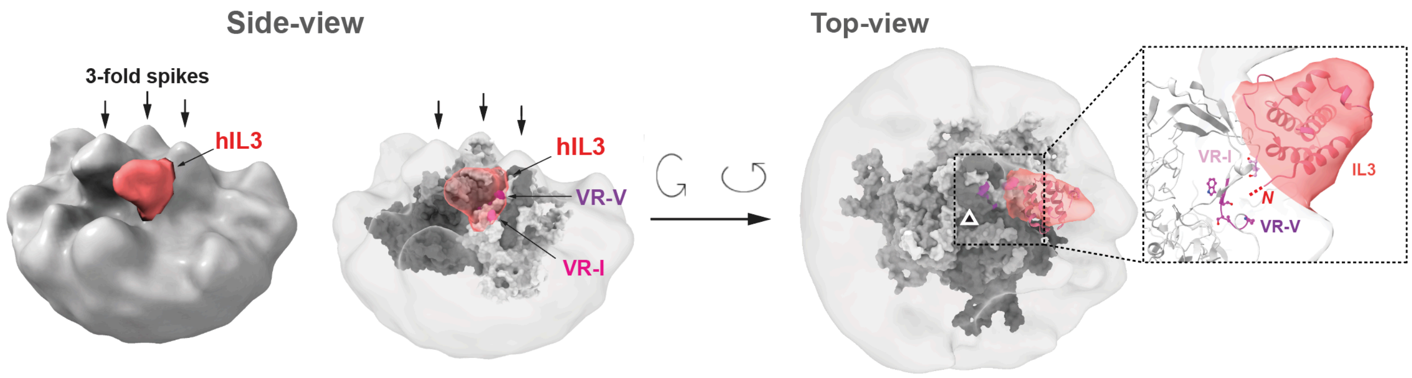

Supplementary Figure 7. Integrative binding model of human IL3-Fc-bound AAV9. Side and top views of the map of the AAV9 trimer bound by IL3-Fc obtained after symmetry expansion and particle subtraction followed by multiple rounds of refinement, with models of the AAV9 trimer (PDB ID: 3UX1) and human IL3 (PDB ID: 5UV8, note truncated N-terminus) docked. Human IL3-Fc density is segmented in red. Capsid variable regions that govern human IL3 binding are indicated (VR-I: pink, VR-V: purple).

Supplementary Figure 8. LRP6 binding to X1 peptide in multiple serotypes and to AAV-BI30

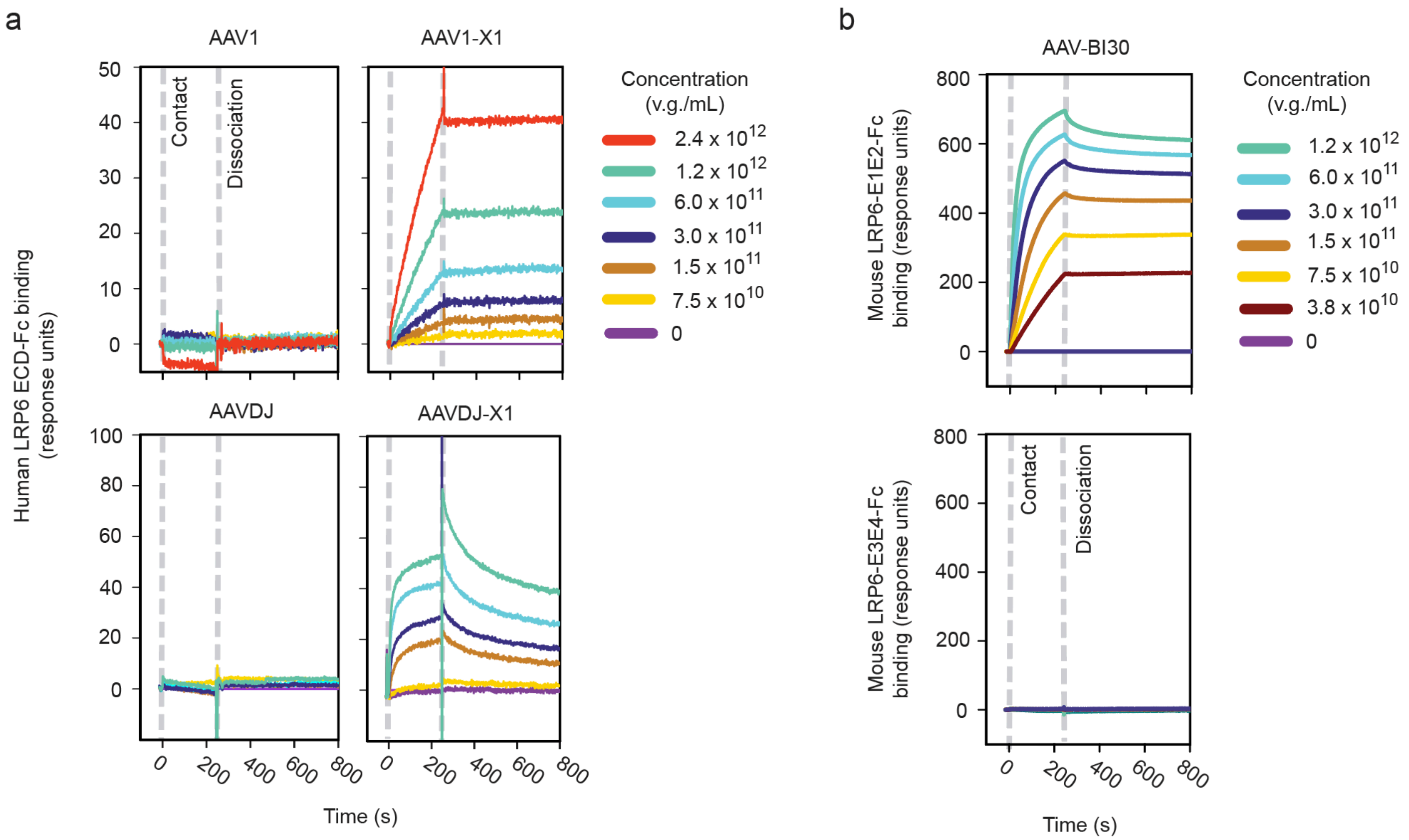

Supplementary Figure 8. LRP6 binding to X1 peptide in multiple serotypes and to AAV-BI30. a SPR of the complete human LRP6 extracellular domain confirms that the X1 insertion peptide modularly enables LRP6 binding across multiple serotypes. **b** SPR of AAV-BI30 confirms binding interaction with mouse LRP6-E1E2 and not LRP6-E3E4. v.g.: viral genomes.

Supplementary Figure 9. Pull-down assay of AAV capsids with known and potential receptors

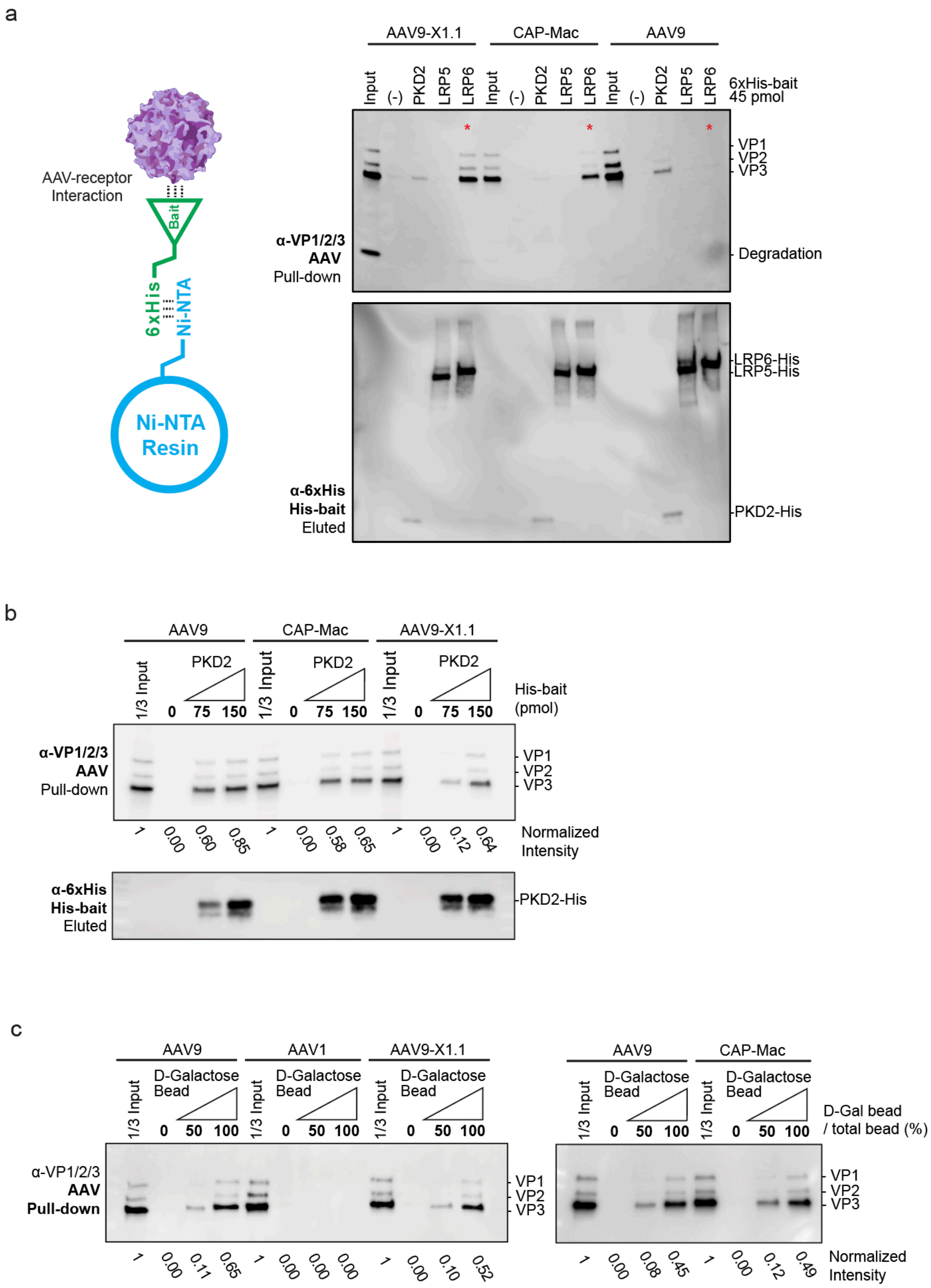

Supplementary Figure 9. Pull-down assay of AAV capsids with known and potential receptors. a *Left*, Schematic of receptor pull-down experimental setup. *Right,* Pull-down assay with the extracellular domains of mouse LRP6, mouse LRP5, and human AAVR PDK2 domain against AAV9, AAV9-X1.1, and CAP-Mac prey. Red asterisks indicate the LRP6 binding interaction gained by X1.1 and CAP-Mac during directed evolution from parent capsid AAV9. **b** Semi-quantitative pull-down assay using human AAVR PKD2 domain bait and AAV9, CAP-Mac, and AAV9-X1.1 prey. Normalized band intensity is quantified beneath each lane. Source data of uncropped blots provided at the end of this file. **c** Semi-quantitative pull-down assay using D-Galactose-coupled resin bait and AAV9, AAV1, AAV9-X1.1, and CAP-Mac prey. AAV9 displays its naturally evolved level of binding while AAV1 control is known to bind a different sugar as its primary receptor. Normalized band intensity is quantified beneath each lane. Source data of uncropped blots provided at the end of this file. Panel a created with BioRender.com released under a Creative Commons Attribution-NonCommercial-NoDerivs 4.0 International license.

Supplementary Figure 10. Cell culture potency assay validation of LRP6 interaction

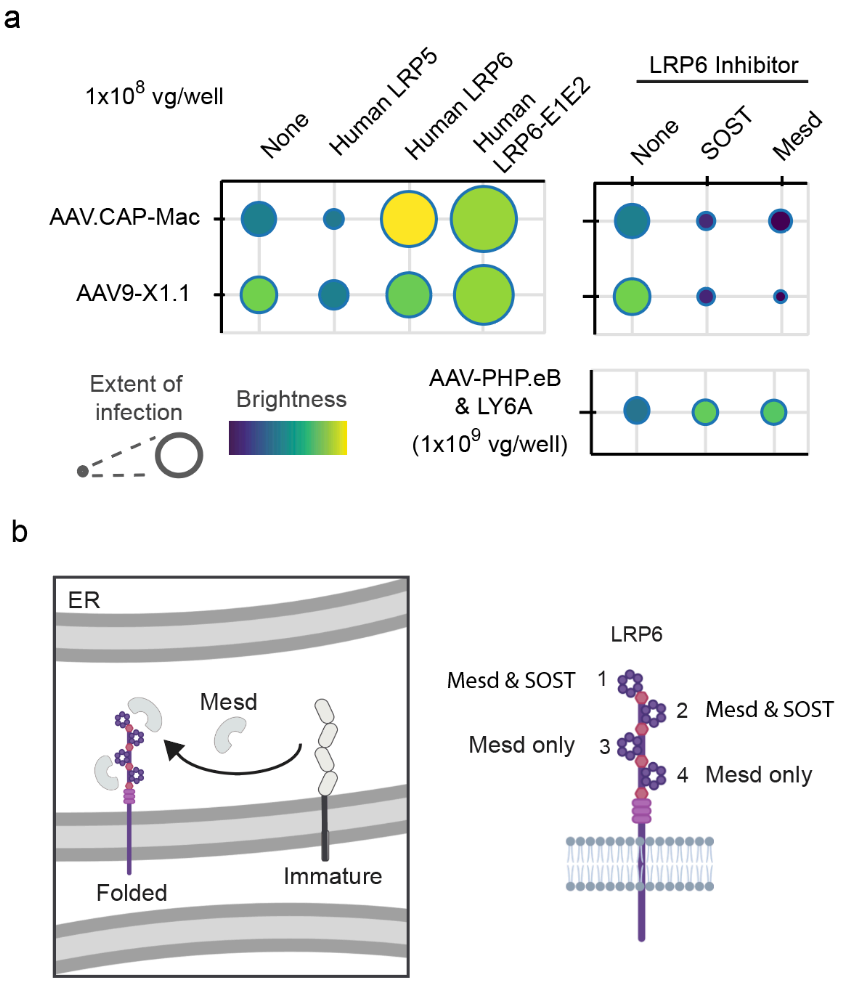

Supplementary Figure 10. Cell culture potency assay validation of LRP6 interaction. a Quantification of AAV potency demonstrating the effects of LRP receptor transient overexpression and LRP6 inhibition. Extent of infection (min, 0.02; max, 0.12) and total brightness per signal area (min, 0.04; max, 0.51). Experiments were performed with 3 biological replicates. v.g.: viral genomes. **b** Schematic of Mesd chaperone function and LRP6 domain-dependent inhibition by recombinant Mesd and SOST proteins. Panel b created with BioRender.com released under a Creative Commons Attribution-NonCommercial-NoDerivs 4.0 International license.

Supplementary Figure 11. Potency of X1 AAVs in mouse liver and human primary cell culture

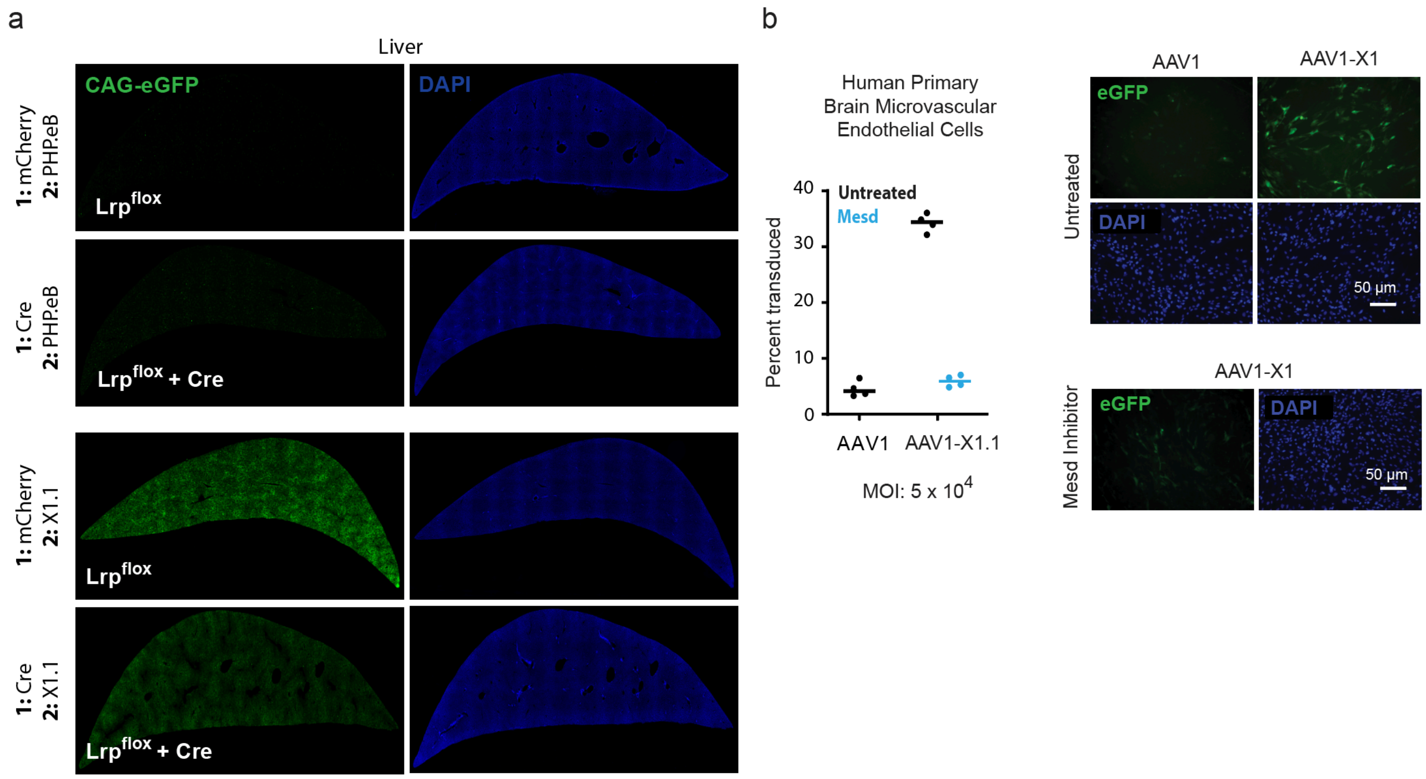

Supplementary Figure 11. Potency of X1 AAVs in mouse liver and human primary cell culture. a AAV9-X1.1 has stronger potency in mouse liver than PHP.eB. Representative liver images of 3 biological replicate animals from Figure 5b with imaging parameters re-optimized for AAV9-X1.1 LRP^{flox} and applied to all conditions. **b** AAV1-X1 has enhanced potency in human primary brain microvascular endothelial cell culture, which decreases to AAV1 levels with Mesd inhibition of LRP6. Bars indicate the mean value. Quantification was performed on 4 biological replicates. MOI: multiplicity of infection.
