# Supplementary material for: Human cell surface-AAV interactomes identify LRP6 as blood-brain barrier transcytosis receptor and immune cytokine IL3 as AAV9 binder
Source: Nat Commun. 2024 Sep 8;15:7853. doi: 10.1038/s41467-024-52149-0 (PMC11381518; doi:10.1038/s41467-024-52149-0)
Supplement: Supplementary file 3 — Reporting Summary [file 41467_2024_52149_MOESM3_ESM.pdf]

nature portfolio

Corresponding author(s): Viviana Gradinaru
Timothy Shay

Last updated by author(s): 08/05/2024

Reporting Summary

Nature Portfolio wishes to improve the reproducibility of the work that we publish. This form provides structure for consistency and transparency in reporting. For further information on Nature Portfolio policies, see our Editorial Policies and the Editorial Policy Checklist.

Statistics

For all statistical analyses, confirm that the following items are present in the figure legend, table legend, main text, or Methods section.

n/a	Confirmed	
☒	☐	The exact sample size (*n*) for each experimental group/condition, given as a discrete number and unit of measurement
☒	☐	A statement on whether measurements were taken from distinct samples or whether the same sample was measured repeatedly
☒	☐	The statistical test(s) used AND whether they are one- or two-sided<br>*Only common tests should be described solely by name; describe more complex techniques in the Methods section.*
☒	☐	A description of all covariates tested
☒	☐	A description of any assumptions or corrections, such as tests of normality and adjustment for multiple comparisons
☒	☐	A full description of the statistical parameters including central tendency (e.g. means) or other basic estimates (e.g. regression coefficient) AND variation (e.g. standard deviation) or associated estimates of uncertainty (e.g. confidence intervals)
☒	☐	For null hypothesis testing, the test statistic (e.g. *F*, *t*, *r*) with confidence intervals, effect sizes, degrees of freedom and *P* value noted<br>*Give P values as exact values whenever suitable.*
☒	☐	For Bayesian analysis, information on the choice of priors and Markov chain Monte Carlo settings
☒	☐	For hierarchical and complex designs, identification of the appropriate level for tests and full reporting of outcomes
☒	☐	Estimates of effect sizes (e.g. Cohen's *d*, Pearson's *r*), indicating how they were calculated

Our web collection on statistics for biologists contains articles on many of the points above.

Software and code

Policy information about availability of computer code

Data collection	For images acquiring and processing, we used Zen Black 2.3 SP1, BZ-X Analyzer 1.4.0.1 and Imaris 9 software. Cryo-ET data were collected using SerialEM. Structural modeling were performed AlphaFold-Multimer-v3 in ColabFold v2.3.5.
Data analysis	Plots were prepared with Graph-Pad Prism 9.0.1 software. Cross-linking mass spectrometry data was analyzed using Proteome Discoverer (v2.5), and xiSPEC. Cryo-ET data analysis was performed in Warp, Dynamo, and Relion (v3.1.3). Structural models were visualized in ChimeraX (v1.6.1) and PyMOL (v2.5.5). Microscopic images were analyzed in Zen Black 2.3 SP1 (Zeiss), ImageJ 1.52q, and Imaris (v9.2.0). Cell potency assay quantification was performed using previously reported Python code available at github.com/GradinaruLab/in-vitro-transduction-assay. Retrogenix cell microarray images were analyzed in ImageQuant.

For manuscripts utilizing custom algorithms or software that are central to the research but not yet described in published literature, software must be made available to editors and reviewers. We strongly encourage code deposition in a community repository (e.g. GitHub). See the Nature Portfolio guidelines for submitting code & software for further information.

Data

Policy information about availability of data

All manuscripts must include a data availability statement. This statement should provide the following information, where applicable:

- Accession codes, unique identifiers, or web links for publicly available datasets
- A description of any restrictions on data availability
- For clinical datasets or third party data, please ensure that the statement adheres to our policy

Cryo-ET data has been deposited to the EMDB with accession codes EMD-42063 (I1 map) [https://www.ebi.ac.uk/pdbe/entry/emdb/EMD-42063] and EMD-41918 (trimer face) [https://www.ebi.ac.uk/pdbe/entry/emdb/EMD-41918]. Cross-linking mass spectrometry data has been deposited to the ProteomeXchange Consortium via the PRIDE84 partner repository with the dataset identifier PXD045380 [http://proteomecentral.proteomexchange.org/cgi/GetDataset?ID=PXD045380]. All other data supporting the findings of this study are provided as source data files. Previously published data used in the present study include: IL3 structure, PDB ID: 5UV8 [http://doi.org/10.2210/pdb5UV8/pdb]; AAV9 structure, PDB ID: 3UX1 [http://doi.org/10.2210/pdb3UX1/pdb].

Human research participants

Policy information about studies involving human research participants and Sex and Gender in Research.

Reporting on sex and gender	*Use the terms sex (biological attribute) and gender (shaped by social and cultural circumstances) carefully in order to avoid confusing both terms. Indicate if findings apply to only one sex or gender; describe whether sex and gender were considered in study design whether sex and/or gender was determined based on self-reporting or assigned and methods used. Provide in the source data disaggregated sex and gender data where this information has been collected, and consent has been obtained for sharing of individual-level data; provide overall numbers in this Reporting Summary. Please state if this information has not been collected. Report sex- and gender-based analyses where performed, justify reasons for lack of sex- and gender-based analysis.*
Population characteristics	*Describe the covariate-relevant population characteristics of the human research participants (e.g. age, genotypic information, past and current diagnosis and treatment categories). If you filled out the behavioural & social sciences study design questions and have nothing to add here, write "See above."*
Recruitment	*Describe how participants were recruited. Outline any potential self-selection bias or other biases that may be present and how these are likely to impact results.*
Ethics oversight	*Identify the organization(s) that approved the study protocol.*

Note that full information on the approval of the study protocol must also be provided in the manuscript.

Field-specific reporting

Please select the one below that is the best fit for your research. If you are not sure, read the appropriate sections before making your selection.

[x] Life sciences [ ] Behavioural & social sciences [ ] Ecological, evolutionary & environmental sciences

For a reference copy of the document with all sections, see nature.com/documents/nr-reporting-summary-flat.pdf

Life sciences study design

All studies must disclose on these points even when the disclosure is negative.

Sample size	No sample size calculation was performed. A sample size of 3 mice or at least 3 biological replicates were chosen for in vivo and in vivo validation experiments. Precise numbers are given in the relevant figure legends and methods sections. Sample sizes were set based on prior experience (Kumar et al Nature Methods 2020, Shay Sullivan Ding et al Sci Adv 2023).
Data exclusions	No data were excluded from the analysis.
Replication	Each in vitro experiment were evaluated in multiple repeats. Details are provided in relevant figure legends and methods sections. Animal experiments used three animals. All attempts at replication were successful.
Randomization	Samples or animals were randomly allocated into experimental group. For imaging and quantification we randomly selected the fields-of-views in the specific regions of the brain.
Blinding	The investigators were not blinded to allocation during group allocation, data collection, or analysis as the differences between control and experimental groups were too obvious and, thus, blinding would not have been practicable.

Reporting for specific materials, systems and methods

We require information from authors about some types of materials, experimental systems and methods used in many studies. Here, indicate whether each material, system or method listed is relevant to your study. If you are not sure if a list item applies to your research, read the appropriate section before selecting a response.

Materials & experimental systems

n/a	Involved in the study
☐	☒ Antibodies
☐	☒ Eukaryotic cell lines
☒	☐ Palaeontology and archaeology
☐	☒ Animals and other organisms
☒	☐ Clinical data
☒	☐ Dual use research of concern

Methods

n/a	Involved in the study
☒	☐ ChIP-seq
☒	☐ Flow cytometry
☒	☐ MRI-based neuroimaging

Antibodies

Antibodies used	Anti-AAV9 clone HL2372 from Merck, Cat# MABF2309-100UL, diluted 1:500<br>Anti-LRP6 from ThermoFisher, Cat# PA5-89161, diluted 1:200<br>Anti-NeuN [1B7] from Abcam, Cat# ab104224, diluted 1:500
Validation	Anti-AAV9 clone HL2372 from Merck, Cat# MABF2309-100UL (https://www.emdmillipore.com/US/en/product/Anti-Adeno-associated-Virus-9-Antibody-clone-HL2372,MM_NF-MABF2309-100UL)<br>Anti-LRP6 from ThermoFisher, Cat# PA5-89161(https://www.thermofisher.com/antibody/product/LRP6-Antibody-Polyclonal/PA5-89161)<br>Anti-NeuN [clone 1B7] from Abcam, Cat# ab104224 (https://www.abcam.com/products/primary-antibodies/neun-antibody-1b7-neuronal-marker-ab104224.html)

Eukaryotic cell lines

Policy information about cell lines and Sex and Gender in Research

Cell line source(s)	HEK293T cells (ATCC, CRL-3216)<br>Human brain microvascular endothelial cells (ScienCell Research Laboratories, Cat# no. 1000), sex not specified<br>Cynomolgus monkey primary brain microvascular endothelial cells (CellBiologics, Cat# MK-6023) , sex not specified<br>Expi293F cells (Thermo Fisher, Cat# A14527)<br>Human embryonic stem line(CSES07) was obtained from Cedars-Sinai Medical Center(NIH approval number: NIHhESC-11-0108)
Authentication	None of the cell lines used were authenticated.
Mycoplasma contamination	The cell lines were not tested for mycoplasma contamination.
Commonly misidentified lines<br>(See ICLAC register)	No cell lines used in this studies are commonly misidentified.

Animals and other research organisms

Policy information about studies involving animals; ARRIVE guidelines recommended for reporting animal research, and Sex and Gender in Research

Laboratory animals	Adult (6-8 weeks old) homozygous B6;129S-Lrp6tm1.1Vari/J mice (Jackson Labs #026267).
Wild animals	The study did not involve wild animals.
Reporting on sex	Both sexes were used across the whole study and the sex information was indicated in the detailed descriptions for each experiments.
Field-collected samples	The study did not involve field-collected samples.
Ethics oversight	All mouse procedures were approved by the California Institute of Technology Institutional Animal Care and Use Committee (IACUC) and comply with all relevant ethical regulations.

Note that full information on the approval of the study protocol must also be provided in the manuscript.
